# Comparative Genomics of Pathogenic and Nonpathogenic Beetle-Vectored Fungi in the Genus *Geosmithia*

**DOI:** 10.1093/gbe/evx242

**Published:** 2017-11-23

**Authors:** Taruna A Schuelke, Guangxi Wu, Anthony Westbrook, Keith Woeste, David C Plachetzki, Kirk Broders, Matthew D MacManes

**Affiliations:** Department of Molecular, Cellular, & Biomedical Sciences, University of New Hampshire; Department of Bioagricultural Sciences and Pest Management, Colorado State University; Department of Computer Science, University of New Hampshire; USDA Forest Service Hardwood Tree Improvement and Regeneration Center, Department of Forestry and Natural Resources, Purdue University

**Keywords:** *Geosmithia morbida*, pathogenicity, tree pathogen, thousand cankers disease

## Abstract

*Geosmithia morbida* is an emerging fungal pathogen which serves as a model for examining the evolutionary processes behind pathogenicity because it is one of two known pathogens within a genus of mostly saprophytic, beetle-associated, fungi. This pathogen causes thousand cankers disease in black walnut trees and is vectored into the host via the walnut twig beetle. *Geosmithia morbida* was first detected in western United States and currently threatens the timber industry concentrated in eastern United States. We sequenced the genomes of *G. morbida* in a previous study and two nonpathogenic *Geosmithia* species in this work and compared these species to other fungal pathogens and nonpathogens to identify genes under positive selection in *G. morbida* that may be associated with pathogenicity. *Geosmithia morbida* possesses one of the smallest genomes among the fungal species observed in this study, and one of the smallest fungal pathogen genomes to date. The enzymatic profile in this pathogen is very similar to its nonpathogenic relatives. Our findings indicate that genome reduction or retention of a smaller genome may be an important adaptative force during the evolution of a specialized lifestyle in fungal species that occupy a specificniche, such as beetle vectored tree pathogens. We also present potential genes under selection in *G. morbida* that could be important for adaptation to a pathogenic lifestyle.

## Introduction

Uncovering the specific genetic and molecular events behind the evolution of novel traits such as pathogenicity in fungal species has been a long-standing objective of pathologists. *Geosmithia* (Ascomycota: Hypocreales), a genus first proposed in 1979 for fungi that were formerly placed in genus *Penicillium* (Pitt 1979), serves a paradigm for examining the processes contributing to the evolution of pathogenicity. *Geosmithia* species are filamentous fungi that most commonly associate with phloeophagous bark beetles (Kolařík et al. 2005, 2011), although some *Geosmithia* fungi, such as *G. eupagioceri* and *G. microcorthyli*, are known to affiliate with ambrosia beetles (Kolařík and Jankowiak 2013). *Geosmithia* species and their beetle associates occupy a variety of hosts, including pines, oaks, junipers, and walnut trees (Kolařík et al. 2007; Kolařík and Kirkendall 2010; Kolařík and Jankowiak 2013). The ecology and diversity of symbiosis between these fungi and their beetle associates is poorly understood, but investigators are beginning to explore such relationships (Kolařík et al. 2007; Kolařík and Jankowiak 2013). Although most species in *Geosmithia* are saprotrophic, two species were recently determined to be pathogenic—*Geosmithia pallida* (Lynch et al. 2014) and *Geosmithia morbida* (Tisserat et al. 2009), on coast live oak (*Quercus agrifolia*) and black walnut (*Juglans nigra*), respectively. However, both of these species live saprophytically in association with bark beetles and other tree hosts. It is still unclear what mechanisms allow these species of *Geosmithia* to be pathogenic to a new host whereas other members of the genus remain saprobes.

*Geosmithia morbida* causes thousand cankers disease (TCD) in *J. nigra* (eastern black walnut). Although no evidence of TCD has been detected in other *Juglans* to date, several species, such as *J. californica*, *J. cinerea*, *J. hindsii*, *J. regia*, are also susceptible to the pathogen (Utley et al. 2013). The fungus is most often vectored into its hosts by *Pityophthorus juglandis*, commonly known as the walnut twig beetle (WTB) (Kolařík et al. 2011). Unusual mortality of *J. nigra* was first noted in Colorado, USA in 2001. Since then, nine western states (CO, WA, OR, ID, NV, UT, CA, NM, and AZ) and seven eastern states (PA, OH, IN, MD, VA, TN, and NC) have reported TCD in one or more locations (Zerillo et al. 2014). This increase in TCD is likely a consequence of the expansion of WTB’s geographic range. WTB was present in only four counties of California, Arizona, and New Mexico in the 1960s, however, as of 2014, the beetle has been detected in over 115 counties in the western and eastern United States (Rugman-Jones et al. 2015).

The origin of this pathogen is not clear. However, it has been hypothesized that *G. morbida* may have undergone a host shift from *J. major* (Arizona black walnut) to a more naïve host, *J. nigra*, because the fungus does not cause disease in the Arizona black walnut, and neither WTB nor *G. morbida* were observed in the native range of *J. nigra* until 2010 (Zerillo et al. 2014). *Juglans nigra* is not indigenous to western United States but was planted throughout the region as an ornamental species. An alternative prediction based on *G. morbida* population genetic data is that the origin of *G. morbida* and WTB are the walnut populations of southern California, where the pathogen has been isolated from both healthy and diseased *J. californica* trees (Zerillo et al. 2014).

Early symptoms of infection by *G. morbida* include yellowing, wilting, and thinning of the foliage followed by branch dieback and tree death within 2–3 years after the initial infestation (Tisserat et al. 2009; Kolařík et al. 2011). Little is known about the specific means *G. morbida* employs for initiating and maintaining the infection, or what benefits, if any, the fungus imparts to the WTB vector. However, previous studies have demonstrated that fungal pathogens that occupy ecological niches similar to *G. morbida* must be capable of enduring and combating toxic host environments used by plants to resist infection. For instance, *Grosmannia clavigera*, a fungal symbiont of the mountain pine beetle (*Dendroctonus ponderosae*), can detoxify metabolites such as terpenoids and phenolics produced by the host as defense mechanisms (DiGuistini et al. 2011).

We recently developed a reference genome of *G. morbida* that consisted of 73 scaffolds totaling 26.5 Mb in length (Schuelke et al. 2016). This genome represents one of the smallest fungal tree pathogen genomes reported to date. Rapid changes in genome size have accompanied dramatic biological changes in newly emerged fungal and oomycete species (Raffaele and Kamoun 2012; Adhikari et al. 2013). In fungi, a link has been observed between genome expansion and evolution of pathogenicity (Raffaele and Kamoun 2012). Genome expansions were associated with parasitism in general and increased pathogenicity and virulence in several fungal lineages (Spanu et al. 2010). Previous genome sequencing of *G. morbida* (Schuelke et al. 2016) showed that this newly emerged fungal pathogen has a smaller genome than several of its closely related nonpathogenic relatives in the Hypocreales. Hence, it is possible that *G. morbida* may have taken an evolutionary path to pathogenicity that has not been characterized previously in plant-associated fungi.

The arrival of new pathogens, frequently referred to as Black Swan events due to their perceived unpredictability, represent a significant threat to native and agriculturally important tree species (Ploetz et al. 2013). Thus, beetle-associated symbionts that have switched to pathogenic lifestyles represent excellent models for investigating the evolution of pathogenicity and its relationship to genome size. Although the genus *Geosmithia* is distributed worldwide, *G. morbida*, and more recently, *G. pallida*, are the first members of the genus to be described as plant pathogens among the 60 known nonpathogenic species (Kolařík and Kirkendall 2010; Kolařík et al. 2011; Lynch et al. 2014).

In this work, we compare the reference genome of the pathogenic and host-specific species *G. morbida* with two closely related nonpathogenic generalist species, *Geosmithia flava* and *Geosmithia putterillii*. Based on this comparison, we identify genes under positive selection that may be involved in the specialization of a pathogenic life strategy that depends on a single beetle vector and a narrow, but potentially expanding, host range. We also present a species phylogeny estimated using single-copy orthologs that confirms the placement of *Geosmithia* species in the order Hypocreales, and that their closest fungal relative is *Acremonium chrysogenum*. The primary goal of this study was to gain insight into the evolution of pathogenicity within *G. morbida*. We also investigated the presence of convergent evolution in *G. morbida* and *G. clavigera*, two tree pathogens vectored into their hosts via beetle symbionts.

## Materials and Methods

### DNA Extraction and Sequencing

The CTAB method delineated by the Joint Genome Institute was used to extract DNA for genome sequencing from lyophilized mycelium of *Geosmithia flava* and *Geosmithia putterillii* (Kohler et al. 2011). Table 1 lists genetic, geographic, and host information for each *Geosmithia* species used in this study. Total DNA concentration was measured with Nanodrop, and DNA sequencing was conducted at Purdue University Genomics Core Facility in West Lafayette, Indiana. DNA libraries were prepared using the paired-end Illumina Truseq protocol and sequenced on an Illumina HiSeq 2500 using a single lane. Mean insert sizes for *G. flava* and *G. putterillii* were 477 and 513 bp, correspondingly. The remaining sequencing statistics are listed in table 2.

**Table 1:** Species, Geographic Origins, and Host Information for *Geosmithia morbida*, *Geosmithia flava*, and *Geosmithia putterillii*

Species	Pathogen	Isolate	Geographic Origins	Host
*G. morbida*^a^	Yes	1262	California	*J. californica*
*G. flava*	No	CCF3333	Czech Republic	*Castenea sativa*
*G. putterillii*	No	CCF4204	California	*J. californica*

a This isolate is the reference genome. The details of assembly for this genome are discussed in Schuelke et al. (2016).

**Table 2:** Statistics for Sequence Data from Isolates of *Geosmithia morbida*, *Geosmithia flava*, and *Geosmithia putterillii*

Species	Total Read Pairs		Est. Coverage	
*G. morbida*	14,013,863^a^	20,674,289^b^	109×^a^	160×^b^
*G. flava*	16,183,281		102×	
*G. putterillii*	19,711,745		131×	

a These values are for paired-end read data for *G. morbida* from Schuelke et al. (2016).

b These values are for mate-pair read data for *G. morbida* from Schuelke et al. (2016).

### Preprocessing Sequence Data

The raw paired-end reads for *G. flava* and *G. putterillii* were corrected using BFC (version r181) (Li 2015). BFC utilizes a combination of hash table and bloom-filter to count *k*-mers for a given read and correct errors in that read based on the *k*-mer support. Because BFC requires interleaved reads as input, khmer 1.1 was leveraged to interleave as well as split the paired-end reads before and after the error correction stage, respectively (Crusoe et al. 2015). Next, low quality bases and adapters in error corrected reads were trimmed with Trimmomatic, version 0.32, using a Phred threshold of 4 (Bolger et al. 2014).

### Assembly Construction

Genome assemblies were constructed with ABySS 1.9.0 using four k-mer sizes (61, 71, 81, and 91) (Simpson et al. 2009). The resulting assemblies were evaluated using BUSCO (v1.1b1) (Simao et al. 2015), a tool which assess completeness based on the presence of universal single-copy orthologs within fungi. Length-based statistics were generated with QUAST v2.3 (Gurevich et al. 2013). Final assemblies were manually chosen based on length-based and genome completeness statistics. Furthermore, the raw reads of *G. flava* and *G. putterillii* were mapped back to their corresponding genomes using BWA version 0.7.9a-r786 (Li and Durbin 2009) to assess the quality of the chosen assemblies.

### Structural and Functional Annotation

We utilized the automated annotation software Maker version 2.31.8 (Cantarel et al. 2008) to functionally annotate the genomes of *G. flava* and *G. putterillii*. We used two of the three gene prediction tools available within the pipeline SNAP (released 2013, Korf 2004) and Augustus 2.5.5 (Stanke et al. 2006). SNAP was trained using gff files generated by CEGMA v2.5 (a program similar to BUSCO) (Parra et al. 2007). Augustus was trained with *Fusarium solani* protein models (v2.0.26) downloaded from Ensembl Fungi (Kersey et al. 2016). The protein sequences generated by the structural annotation were blasted against the Swiss-Prot database (Boutet et al. 2016) to functionally annotate the genomes of *G. flava* and *G. putterillii*. We assessed the completeness of the final transcript sets using BUSCO (v1.1b1) and fungal data set 9 provided with this software.

### Assessing Repetitive Elements Profile

To evaluate the repetitive elements profile of *G. flava* and *G. putterillii*, we masked the interspersed repeats within the assembled genomes with RepeatMasker 4.0.5 (Smit et al. 1996) using the sensitive mode and default values as arguments.

In most fungi, repeat-induced point mutations are a defense mechanism against the propogation of repetitive genetic elements and tend to favor the conversion of cytosines to thymines resulting in AT-rich regions (Selker 2002). Therefore, we also examined whether *Geosmithia* species have undergone repeat-induced point mutations using OcculterCut v1.1 (Testa et al. 2016).

### Identifying Putative Genes Involved in Host–Pathogen Interactions

To search for putative genes contributing to pathogenicity, we conducted a BLASTp (v2.2.28+) (Altschul et al. 1990) search with an e-value threshold of 1e–6 against the PHI-base 4.0 database (Winnenburg et al. 2006) that includes known genes implicated in pathogenicity. We also assessed how many of the peptides with PHI-base hits were cysteine-rich proteins based on methods described in Kim et al. (2016). A protein was considered cysteine-rich if the length was 300 or less amino acids and the cysteine percentage was at least 3% of the total length. Further, we identified proteins that contain signal peptides and lack transmembrane domains in each *Geosmithia* species as well as their close relative *A. chrysogenum* with SignalP 4.1 and TMHMM 2.0 using default parameters (Krogh et al. 2001; Peterson et al. 2011).

### Identifying Species-Specific Genes

To identify unique genes present in *G. morbida*, we performed an all-versus-all BLASTp search among the three *Geosmithia* species and *A. chrysogenum* with Orthofinder version 0.3.0 (Emms and Kelly 2015). Using a custom Python script, we analyzed homology among the four fungal species.

### Identifying Carbohydrate-Active Proteins and Peptidases

To identify enzymes capable of degrading carbohydrate molecules in species belonging to Hypocreales and *G. clavigera*, we performed a HMMER 3.1b1 (Eddy 1998) search against the CAZy database (Lombard et al. 2014) released July 2015 and filtered the results following the developer’s recommendations. Finally, we profiled the proteolytic enzymes present in species using the *MEROPS* database 10.0 (Rawlings et al. 2016).

### Identifying Secondary Metabolite Biosynthesis Gene Clusters

To identify gene clusters involved in secondary metabolite biosynthesis, we used antiSMASH 3.0 (Weber et al. 2015) to locate such gene clusters in the genomes. The analysis was conducted with default settings with the “DNA of eukaryotic origin” option.

### Phylogenetic Analysis

#### Taxon Sampling

In order to determine phylogenetic position of *Geosmithia*, we combined the predicted peptide sequences from three *Geosmithia* species described here with the predicted peptide sequences of an additional 17 fungal genomes that represent the breadth of pathogens and nonpathogens within Ascomycota. Our data set contained eleven pathogens and nine nonpathogens (table 3).

**Table 3:** Fungal Species Used for Phylogenetic Analysis in This Study

Species	Class	Order	Ecological Role	Download Source	References
***G. morbida***	Sordariomycetes	Hypocreales	Pathogen	—	Schuelke et al. (2016)
***G. flava***	Sordariomycetes	Hypocreales	Nonpathogen	—	–
***G. putterillii***	Sordariomycetes	Hypocreales	Nonpathogen	—	–
***A. chrysogenum***	Sordariomycetes	Hypocreales	Beneficial	FungalEnsembl	Terfehr et al. (2014)
***S. grisellum***	Sordariomycetes	Hypocreales	Saprotrophic	JGI	Used with permission
*Trichoderma virens*	Sordariomycetes	Hypocreales	Mycoparasite	JGI	Kubicek et al. (2011)
***Trichoderma reesei***	Sordariomycetes	Hypocreales	Saprotrophic	FungalEnsembl	Martinez et al. (2008)
*Escovopsis weberi*	Sordariomycetes	Hypocreales	Mycoparasite	EnsemblGenomes	de Man et al. (2016)
*Ustilaginoidea virens*	Sordariomycetes	Hypocreales	Biotrophic pathogen	FungalEnsembl	Zhang et al. (2014)
*C. militaris*	Sordariomycetes	Hypocreales	Insect pathogen	FungalEnsembl	Zheng et al. (2011)
***M. inundatum***	Sordariomycetes	Hypocreales	Saprotrophic	JGI	Used with permission
*F. solani*	Sordariomycetes	Hypocreales	Necrotrophic pathogen	FungalEnsembl	Coleman et al. (2009)
*Fusarium graminearum*	Sordariomycetes	Hypocreales	Necrotrophic pathogen	FungalEnsembl	Trail et al. (2003), Cuomo et al. (2007) and Ma et al. (2010)
*C. platani*	Sordariomycetes	Microascales	Pathogen	FungalEnsembl	Belbahri (2015)
***Neurospora crassa***	Sordariomycetes	Sordariales	Saprotrophic	FungalEnsembl	Galagan et al. (2003)
***Chaetomium globosum***	Sordariomycetes	Sordariales	Saprotrophic	JGI	Berka et al. (2011)
***Grosmannia clavigera***	Sordariomycetes	Ophiostomatales	Pathogen	FungalEnsembl	DiGuistini et al. (2011)
*Eutypa lata*	Sordariomycetes	Xylariales	Pathogen	JGI	Blanco-Ulate et al. (2013)
*Botrytis cinerea*	Leotiomycetes	Helotiales	Necrotrophic pathogen	FungalEmsebl	Amselem et al. (2011) and Staats and van Kan (2012)
***Oidiodendron maius***	Leotiomycetes	Incertae sedis	Mycorrhizal	JGI	Kohler et al. (2015)

Note.—The species in bold were utilized for positive selection analysis.

#### Inferring Orthology

Orthologous peptide sequences among the 20 fungal genomes were determined using OrthoFinder version 0.3.0 (Emms and Kelly 2015). All-versus-all BLASTp (2.2.28+, Altschul et al. 1990) searches were performed among a set of protein coding genes to infer orthogroups and aligned using MAFFT (v7.123b, Katoh and Standley 2013). These orthogroups may contain paralogs as well as orthologs, and because data sets rich in paralogs can confound phylogenomic analysis, the orthogroup alignment files produced by OrthoFinder were parsed to recover only those that contained single-copy orthologs from each of the 20 species. This resulted in 1,916 total orthogroups with 100% taxon occupancy.

#### Trimming Alignments

For each alignment, regions that contained gap rich sites were removed using *–gappyout* option in trimAl v1.4.rev15 (Capella-Gutiérrez et al. 2009). Next, all files containing orthogroups were renamed so the respective headers among these files were identical and individual alignments were concatenated. Concatenation resulted in a fasta file containing all 1,916 partitions with 1,054,662 amino acid sites at 100% taxon occupancy. This initial alignment was further filtered using MARE (v.0.1.2) (Misof et al. 2013), which reduced the data matrix to 633 partitions and 247,627 sites. Next, the best-fit substitution models for each partition and a global partitioning scheme were determined with PartitionFinder (v1.1.1) using hcluster clustering algorithm and default parameters (Lanfear et al. 2014).

#### Constructing Phylogeny

Maximum likelihood (ML) analysis was conducted in RaxML v 8.1.20 (Stamatakis 2014) leveraging the partitioning scheme determined by PartitionFinder. The ML tree and 200 bootstrap replicates were performed in a single analysis using the *–f a* option. In addition, we estimated the phylogenies of each of the 633 individual partitions under the LG model and used these topologies to estimate internode certainty (IC) and tree certainty (TC) measures (Salichos et al. 2014). Bayesian Markov Chain Monte Carlo (BMCMC) analysis was performed in MrBayes 3.2.6 (Ronquist et al. 2012) and PhyloBayes 4.1 (Lartillot et al. 2009). For MrBayes analysis, we truncated the alignment to contain the maximum number of positions allowed in MrBayes (89,999 sites), specified the mixed amino acid model prior and, based on preliminary analyses of larger data sets (not shown), ran the tree search for 215,000 generations. After testing for convergence, a consensus tree was generated after discarding 25% of the run as burnin using the sumt command. The nexus file, including MrBayes block, provides other details of the MrBayes analysis (supplementary Methods S1, Supplementary Material online). PhyloBayes analyses were performed under the CAT-GTR model and the posterior distributions of two independent runs were assessed with a burn-in of 1,000 and capturing every 2 trees using the bpcomp program. The complete data matrix and scripts used in these analyses can be found in our online repository.

#### Detecting Genes under Positive Selection

To identify genes under positive selection in *G. morbida*, we compared *G. morbida* with all nonpathogens from the aforementioned 20 fungi used to estimate the species tree. Among this batch of 10 fungal species, we detected 22,908 protein orthogroups using OrthoFinder that contained paralogs as well as orthologs. Of these, only 9,560 orthogroups were alignable with MAFFT because many groups consisted of only one sequence from a single species (Katoh and Standley 2013). A total of 3,327 orthogroups, composed of single-copy orthologs, were filtered and corresponding coding DNA sequences for each peptide in these partitions were extracted using custom scripts that can be found online.

The coding DNA sequences were then aligned with MACSE v1.01.b (Ranwez et al. 2011). This Java-based utility accounts for frameshifts and premature stop codons in coding sequences during the alignment process and outputs aligned protein and nucleotide sequences. In order to filter out alignments with frameshifts and internal stop codons, we utilized a program called PAL2NAL v14 (Suyama et al. 2006). This software searches for complementary regions between multiple protein alignments and the corresponding coding DNA sequences, and omits any problematic codons from the output file. This cleaning step reduced the number of 3,327 orthogroups to 2,798 that were used for detecting genes under selective pressures.

The branch-site model (BSM) in the CodeML program of package PAML v4.8 was used for selection analysis (Yang 2007). BSM permits ω (d*N*/d*S*) to vary among sites and branches permitting the identification of specific branches and sites subjected to selection. We computed two models in order to calculate and compare the likelihood values: a null model with a fixed ω value of 1 and an alternative model that estimates ω in the foreground branch, which is *G. morbida* in our case. In the effort to reduce false positives, we implemented the Benjamini–Hochberg correction method when comparing likelihood ratios for null and alternative models using a *P*-value threshold of 0.05. We performed similar BLAST searches as mentioned previously to characterize the functions of these proteins and identify proteins with signal peptides and transmembrane domains.

The above procedures were repeated for detecting genes under selection in *G. clavigera* because this fungal pathogen plays an ecological role similar to *G. morbida*. By performing these analyses, we sought to uncover genes under adaptive evolution in both beetle-vectored tree pathogens.

## Results

### Assembly Features

We recently assembled a reference genome for a *G. morbida* strain isolated from *J. californica* in Southern California (Schuelke et al. 2016). The reference contained 73 scaffolds with an estimated size of 26.5 Mb. By using the MAKER annotation pipeline, we predicted 6,273 protein models in this reference in-silico (Cantarel et al. 2008). In this work, we sequenced strains of *G. flava* and *G. putterillii* at approximately 102× and 131× coverage, respectively. The *G. flava* assembly was composed of 1,819 scaffolds totaling 29.47 Mb in length, and the *G. putterillii* genome contained 320 scaffolds extending 29.99 Mb. *Geosmithia flava* and *G. putterillii* totaled 6,976 and 7,086 protein models, respectively. Both genomes contained 98% of the single-copy orthologs present in more than 90% of the fungal species. Nearly all of the raw reads (97% and 98%) mapped back to *G. flava* and *G. putterillii* genome assemblies, respectively (table 4). These statistics indicated that our genome assemblies are high quality and complete.

**Table 4:** Length-Based Statistics for *Geosmithia morbida*, *Geosmithia flava*, and *Geosmithia putterillii* Generated with QUAST v2.3

Species	Est. Genome Size (Mb)	*k*-mer for ABySS Assembly	Scaffold Count	Largest Scaffold	NG50	LG50	Genome Completeness	Predicted Proteins	Transcript Completeness^b^
*G. morbida*	26.5	NA^a^	73	2,597,956	1,305,468	7	98	6,273	93
*G. flava*	29.6	91	1,819	1,534,325	460,430	22	98	6,976	94
*G. putterillii*	30.0	91	320	2,758,267	1,379,352	9	98	7,086	94

Note.—The average GC content for *G. morbida*, *G. flava*, and *G. putterillii* equals 54%, 52%, and 55.5%, respectively. All genome completeness values were produced with BUSCO v1.1b1. These percentages represent genes that are complete and not duplicated or fragmented. NG50 is the scaffold length such that considering scaffolds of equal or longer length produce 50% of the bases of the reference genome. LG50 is the number of scaffolds with length NG50.

a Genome assembly for *G. morbida* was constructed using AllPaths-LG (v49414). See Schuelke et al. (2016) for further details.

b These percentages were computed using the fungal data set 9 provided with BUSCO.

An estimated 0.80% of *G. morbida* reference genome sequence represented repeats, whereas 0.63% and 0.64% of the sequences in *G. flava* and *G. putterillii* consisted of repetitive elements. There were 60, 42, and 15 DNA transposons in *G. morbida*, *G. flava*, and *G. putterillii*, respectively. Furthermore, *G. morbida* possessed only 152 retroelements, whereas *G. flava* and *G. putterillii* had 401 and 214 of such elements, correspondingly (table 5).

**Table 5:** Repetitive Elements Profile of *Geosmithia* Species Generated with RepeatMasker v4.0.5

	Genome Size (Mb)	GC (%)	Bases Masked (%)	No. of Retroelements	No. of DNA Transposons
*G. morbida*	26.5	54.30	0.81	152	60
*G. flava*	29.6	51.87	0.63	401	42
*G. putterillii*	30.0	55.47	0.64	214	15

We also looked for the presence of repeat-induced point mutations within each *Geosmithia* species by searching for AT-rich regions. In many fungi, AT-rich regions are associated with such mutations that favor the transition of cytosines to thymines resulting in a lower GC content. We did not find AT-rich regions in any of the three *Geosmithia* species (supplementary figs. S2–S4, Supplementary Material online).

### Identifying Putative Genes Involved in Pathogenicity

Approximately 32%, 34%, and 35% of the total proteins in *G. morbida*, *G. flava*, and *G. putterillii* respectively shared significant homology with protein sequences in the PHI-base 4.0 database. The number of unknown proteins with hits in the PHI-base database was similar for *G. morbida* (26), *G. flava* (28), and *G. putterillii* (36). The full BLASTp search results against the PHI-base database for *G. morbida*, *G. flava*, and *G. putterillii* are available in the supporting material (supplementary table S1, Supplementary Material online). Only 20 proteins with PHI-base hits were cysteine-rich peptides in all three species.

### Identifying Species-Specific Genes

An analysis of orthologous groups (OGs) between the three Geosmithia species and *A. chrysogenum* was completed to determine whether a significant loss in OGs in any of the *Geosmithia* species has taken place. The three *Geosmithia* species and *A. chrysogenum* contained a total of 9,065 OGs. Among the set of homologous genes, there were 4,655 single copy orthologs. *A. chrysogenum* contained 2,338 species-specific genes, of which seven genes were paralogous. *Geosmithia morbida* possessed 73 unique genes whereas, *G. putterilli* and *G. flava* had 161 and 146 species-specific genes. The two nonpathogenic *Geosmithia* species did not contain any paralogs, however *G. morbida* had three unique genes present in five copies. Based on a functional search against NCBI’s nonredundant database, the three genes encode hydantoinase B/oxoprolinase, aldehyde dehydrogenase, and ABC-2 type transporter.

A total of 205 OGs were present in *A. chrysogenum*, *G. putterillii*, and *G. flava*, but not in *G. morbida*. This is likely due to a species-specific gene loss events in *G. morbida*, as it was the most parsimonious scenario. At the same time, only 90 and 88 OGs are specifically missing in *G. putterillii* and *G. flava*, respectively. This suggests that *G. morbida* has experienced a higher OG loss rate than the other two *Geosmithia* species.

### Identifying Putative Secreted Proteins

A total of 349, 403, and 395 proteins in *G. morbida*, *G. flava*, and *G. putterillii* contained signal peptides, respectively. Of these putative signal peptide-containing proteins in *G. morbida*, 27 (7.7%) encoded proteins with unknown function, whereas *G. flava* and *G. putterillii* contained 29 (7.2%) and 30 (7.6%) unknown proteins, respectively. The difference in percentage of unknown proteins with signal peptides was minimal among the three genomes. For each species, proteins containing signal peptides were subjected to a membrane protein topology search using TMHMM v2.0. There were 237, 281, and 283 proteins in *G. morbida*, *G. flava*, and *G. putterillii* that lacked any transmembrane protein domains.

### Profiling Carbohydrate-Active Enzymes and Peptidases

**Fig. 1.**
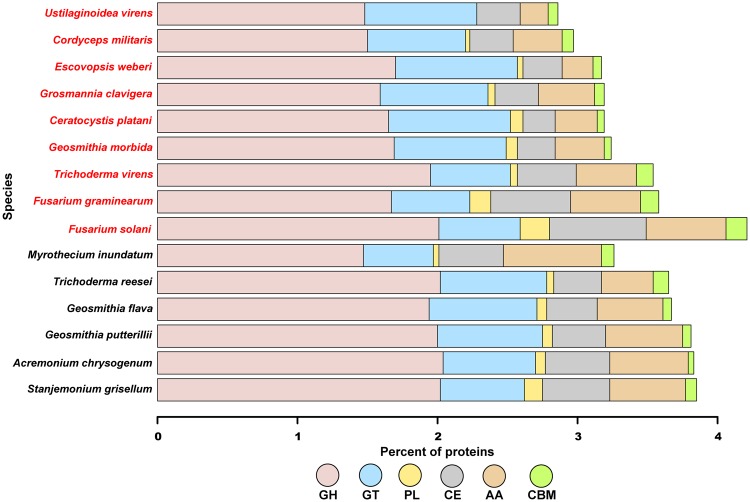
—CAZymes distribution for *Geosmithia* species, other Hypocreales, and *C. platani*. The species in red are pathogens, whereas the names in black are nonpathogens. CAZymes were identified with HMMer searches of dbCAN peptide models. GH, glycoside hydrolases; GT, glycosylTransferases; PL, polysaccharide lyases; CE, carbohydrate esterases; AA, auxiliary activities enzymes; CBM, carbohydrate-binding molecules.

Carbohydrate-active enzymes (CAZymes) break down plant structural components, enabling initiation and establishment of infection. We assessed the CAZymatic profile of all species in the order *Hypocreales*, *Geosmithia* species, and *G. clavigera* (fig. 1). The glycoside hydrolase (GH) family members dominated all protein models, followed by glycosyltransferase (GT) family. The two most prominent families among all fungal species were GH3 and GH16 (supplementary table S2, Supplementary Material online). GH3 hydrolases are involved in cell wall degradation and overcoming the host immune system, and GH16 enzymes fulfill a wide range of cellular functions including transporting amino acids. The third most representative family was GH18; however *G. morbida* only contained four of these enzymes. In contrast, this number for other species ranges from 9 to 31 enzymes. Along with acetylglucosaminidases, family GH18 harbors chitinases that assist in the production of carbon and nitrogen. In terms of other CAZyme families, all fungi except *F. solani* have a similar overall distribution. *Fusarium solani* contains more CAZymes than any other pathogen or nonpathogen.

**Fig. 2.**
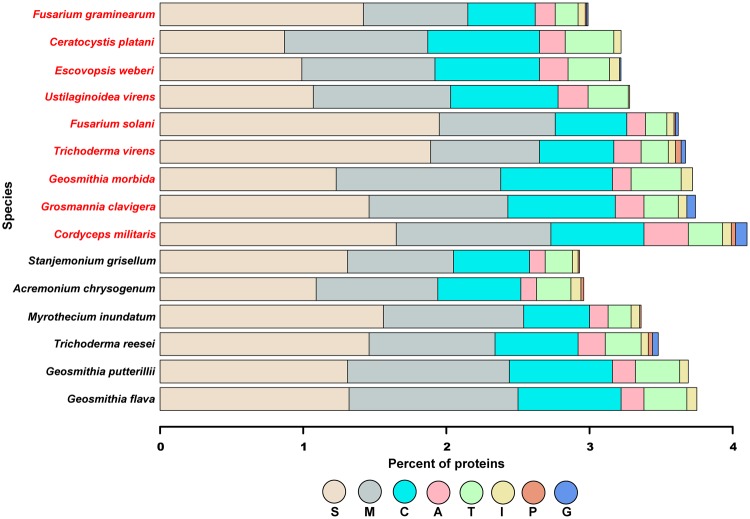
—Proteolytic enzymes distribution for *Geosmithia* species, other Hypocreales, and *C. platani*. The species in red are pathogens, whereas the names in black are nonpathogens. Proteases were identified using BLASTp searches against the MEROPs database v10. S, serine; M, metallo; C, cysteine; A, aspartic; T, threonine; I, inhibitors; P, mixed; G, glutamic.

In addition to profiling CAZymes, we performed a BLAST search against the peptidase database—Merops v10.0 (Rawlings et al. 2016)— for each *Hypocreales*, *Ceratocystis platani*, and *G. clavigera*. Among the pathogens, *G. morbida* has the third highest percent of predicted proteases after *Cordyceps militaris* (insect pathogen) and *G. clavigera* (fig. 2 and supplementary table S3, Supplementary Material online). Moreover, *G. flava* and *G. putterillii* have the largest percent of peptidases among the nonpathogenic fungi. All three *Geosmithia* species illustrate similar proteolytic profiles and contain no glutamic and mixed peptidases.

### Smaller Repertoire of Secondary Metabolite Biosynthesis Gene Clusters in *Geosmithia* Species

Contrary to primary metabolites, secondary metabolites are not necessary for normal fungal growth and development (Fox and Howlett 2008). Although their role is not completely known, it is most likely they help the organism survive its ecological niche (Fox and Howlett 2008). To examine the capacity for secondary metabolite biosynthesis in *Geosmithia* and two most closely related species, we identified gene clusters responsible for such biosynthesis using antiSMASH (Weber et al. 2015). Although the two closely related species, *Acremonium chrysogenum* and *Stanjemonium grisellum*, have large repertoires of such gene clusters (42 and 60, respectively), the *Geosmithia* species have much smaller repetoires, ranging from 14 to 19 gene clusters (supplementary table S4, Supplementary Material online). Within the *Geosmithia* genus, *G. morbida* has an even smaller number of such gene clusters than *G. putterilli* and *G. flava* (14 vs 16 and 19, supplementary table S4, Supplementary Material online).

Furthermore, to assess the impact of a fragmented genome on clusters detection, we plotted N50 values of these genomes against the total cluster numbers. We found no evidence of a positive correlation between the two, indicating that a smaller N50 does not lead to under-detection of gene clusters (supplementary fig. S1, Supplementary Material online).

### Inferring Phylogeny

**Fig. 3.**
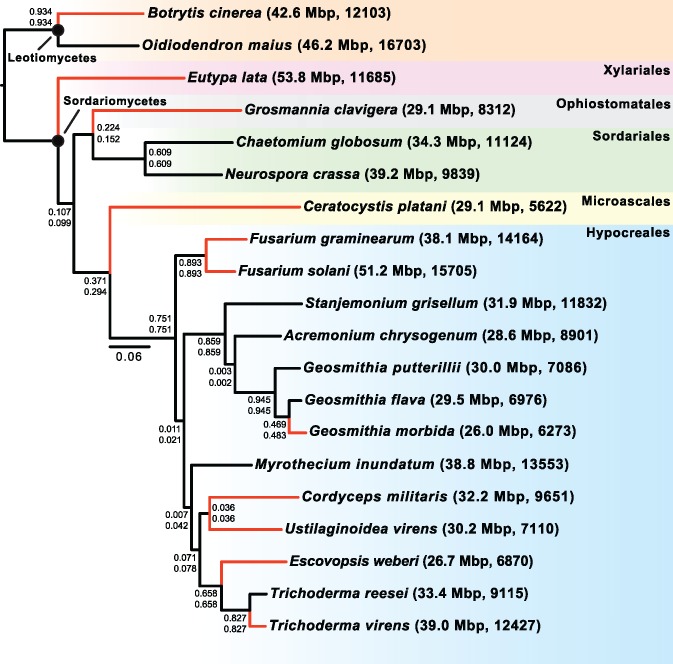
—ML was estimated with RAxML (Stamatakis 2014) using a scheme determined by PartitionFinder (Lanfear et al. 2014). The IC (top) and IC All (bottom) scores are also presented for each node. This topology is identical to the BMCMC phylogeny constructed in MrBayes (Ronquist et al. 2012). All nodes in ML and BMCMC analyses receive maximum support of 1. The black circles symbolize classes. The color-shaded boxes at the right of the figure denote the orders within each class. The first and second numbers in parentheses represent the genome sizes in Mb and the number of predict protein models, respectively. Black and red branches correspond to nonpathogens and pathogens, respectively, which span multiple orders.

Even though *Geosmithia* was first established as a genus in 1979, it has only recently been described in depth. One of the objectives in this study was to uncover the phylogenetic relationship between *Geosmithia* species and other fungal pathogens using coding DNA sequence data. In order to determine the broader evolutionary history of *Geosmithia* species, we constructed ML and BMCMC phylogenies using genome-scale data from *G. morbida*, *G. putterillii*, *G. flava*, and 17 additional fungal taxa (table 3). Our data set consisted of 11 pathogens and 9 nonpathogens. After trimming and filtering, our data matrix comprised of 633 partitions and 247,627 amino acid sites. The topologies generated under ML and BMCMC were identical, and all nodes in both analyses received bootstrap support of 100% (ML) and posterior probabilities of 1.0 (BMCMC). Our MrBayes analysis reached convergence after 215,000 generations with the average standard deviation of split frequencies = 0.000000. Phylobayes analyses had a max diff score of 0.178571 after 2,061 cycles. However, analyses using the standard IC/TC algorithm (Salichos et al. 2014) in RAxML 8.1.2 (Stamatakis 2014) produced a TC value of only 0.457, indicating a significant degree of discordance among topologies for individual partitions. In addition, IC and IC All scores reveal a number of poorly recovered internodes among topologies individual partitions (fig. 3). Taken together, our phylogenomic analyses provide strong support for the monophyly of the *Hypocreales* and the position of *Geosmithia* within it and is consistent with previous reports (Fitzpatrick et al. 2006, Wang et al. 2009). The *Geosmithia* species form a well-supported monophyletic clade with two nonpathogenic fungi, *A. chrysogenum* and *S. grisellum*, suggesting that the common ancestor shared among these species was likely not a pathogen.

### Genes under Positive Selection

In order to understand the molecular basis of pathogenicity in *G. morbida*, we sought to detect genes under positive selection by leveraging the BSM in PAML’s codeml program (4.8). *Geosmithia morbida* was selected as the foreground branch. Our results showed 38 genes to be under positive selection using an adjusted *P*-value < 0.05. Next, we performed a functional search for each protein by blasting the peptide sequences against the NCBI nonredundant and pfam databases. We determined that several were involved in catabolic activity, gene regulation, and cellular transport (table 6 and supplementary table S4, Supplementary Material online).

**Table 6:** Functional Analyses of Genes under Positive Selection in *Geosmithia morbida* Detected by the Branch-Site Model in PAML 4.8

Gene Number	Function	d*N*/d*S*	Transmembrane Domain (*N*)
3078	Takes part in intracellular signaling, protein recruitment to various membranes	2.04	0
2666	Involved in receptor-mediated endocytosis and vesicle trafficking	2.01	0
563	Unclear function	1.94	1
2194	Unknown function	1.94	0
801	Catalyzes the transfer of electrons from ferrocytochrome c to oxygen converting the cytochrome c into water	1.93	1
3944	Involved in methylation and have a wide range of substrate specificity	1.90	5
5058	Involved in ubiquitination of proteins target for degradation	1.90	0
1843	Involved in heat-shock response	1.86	0
521	Involved in damage DNA binding and repair	1.85	0
5111	Involved in receptor-mediated endocytosis and vesicle trafficking	1.84	0
4128	Catalyzes the hydrolysis of esters	1.84	0
923	Hydrolases the peptide bond at the C-terminus of ubiquitin	1.83	1
4405	Involved in transport and metabolism of lipids	1.83	1
3137	Part of proteins with diverse functions such as cell-cycle regulators, signal transducers, transcriptional initiators	1.78	0
4359	Unknown function	1.73	2
5639	Involved in rRNA synthesis	1.67	0
5	Involved in vesicular transport	1.63	0
624	Involved in transfer of glucose molecules that are part of a larger glycosylation machinery	1.62	9
3929	Unknown function but associates with GRAM domain found in glucosyltransferases and other membrane affiliated proteins	1.61	0
1456	Involved in DNA repair and replication	1.59	0
4829	Form cAMP	1.59	0
254	Major ATP transporters	1.59	2
4888	Unknown function	1.54	0
5426	Hydrolyzes nonubiquitinated peptides	1.54	0
5709	Transcription factors	1.50	0
859	May be involved in the timing of nuclear migration	1.50	0
5703	Cleave peptide bonds in other proteins	1.47	6
5255	Heat shock protein involved in induced stress response to ethanol	1.46	3
5704	Regulates gene expression during oxidative stress caused by the host plant	1.46	0
2485	Transfer phosphates	1.39	0
6116	Hydratase and/or isomerase	1.38	0
5266	Breaks down actin, cell membrane deformations	1.34	0
5000	Catalyzes the first step in histidine biosynthesis	1.34	0
3326	Involved in de novo synthesis of nucleotide purine	1.32	0
2142	E2 enzymes that catalyze the binding of activated ubiquitin to the substrate protein. The substrate proteins are targeted for degradation by the proteasome	1.24	0
581	Ribosomal protein	1.17	0
5948	Involved in initiation of transcription	1.14	1
3700	Part of the TOM complex that recognizes and regulates the transport of mitochondrial precursor molecules from the cytosol to the intracellular space of the mitochondrion	1.03	0

Note.—The gene number corresponds to the sequence ID in the *G. morbida* protein file available at DRYAD. The *P*-values for each d*N*/d*S* ratio is < 0.05. d*N*/d*S* is the ratio of nonsynonymous substitutions to synonymous changes.

### Genes under Adaptive Evolution in Beetle-Vectored Fungal Pathogens

In addition to detecting genes under selective pressures in *G. morbida*, we performed the same selection analysis for *Grosmannia clavigera* to identify overlapping proteins that may help explain adaptations leading to the ecological role these two beetle-vectored fungi play. We found that *G. clavigera* possessed 42 positively selected genes that shared protein domains with only two of the 38 genes predicted to be under selection in *G. morbida*. The two overlapping motifs are methyltransferase and protein kinase domains. Our KEGG analysis exhibited no common pathways between *G. morbida* and *G. clavigera*. The complete table displaying BLASTp results for genes under positive selection in *G. clavigera* is available in the supporting material (supplementary table S6, Supplementary Material online).

## Discussion

This study aims to provide insight into the evolution of pathogenicity within *G. morbida*, a beetle vectored pathogen that is the causal agent of TCD in *Julgans* species. Our results indicate that the genome size, gene number, transposon number, and secondary metabolite profile of both pathogenic and nonpathogenic species of *Geosmithia* are very similar. This is not specifically unexpected as all three species occupy similar niches as beetle-vectored fungi associated with trees. The only difference in life history strategy is that *G. flava* and *G. putterilli* have a much large host range and are primarily saprotroph that exist on dead or dying trees including coniferous and deciduous species, whereas *G. morbida* has a pathogenic life strategy and primarily infects members of the genus *Juglans*. The main distinction between *G. morbida* and the nonpathogenic species amounts to 38 genes found to be under positive selection in the former, of which several have known function in pathogenicity in other plant pathogenic fungi. Although small in numbers, it may be these genes that separate the pathogenic *G. morbida* from its saprotrophic congeners.

### The Smaller Genome Size of *Geosmithia Species*

In contrast to other species in the phylogeny (fig. 3), fungi associated with trees either as pathogens or saprophytes (*Geosmithia* species, *G. clavigera*, and *C. platani*) had reduced genomes and gene content. We predict this smaller genome and gene content could potentially be a result of evolving specialized lifestyles to occupy a specialized niche. For instance, all three *Geosmithia* species and *G. clavigera* are vectored into their respective hosts via bark beetles, which may result in strong selection on the genetic variability of the fungi because they must adapt to their vectors and hosts simultaneously. A recent study characterizing the genome of mycoparasite *Escovopsis weberi* showed that specialized pathogens tend to have smaller genomes and predicted protein sets because they lack genes that are not required beyond their restricted niche when compared to closely related generalists (de Man et al. 2016). Our results agree with this finding because *G. morbida* has a more specialized beetle vector (*P. juglandis*) and plant host range (*Juglans* species) in comparison to *G. putterilli* and *G. flava* which can be found on a variety of trees species including both gymnosperms and angiosperms (Kolařík and Jankowiak 2013), and can be vectored by multiple beetle species. Both *G. morbida* and *E. weberi* represent a significant contrast to previous reports that have documented the importance of genome expansion with the evolution of pathogenicity (Adhikari et al. 2013; Raffaele and Kamoun 2012). Furthermore, our results are supported by prior findings which showed that gene loss and gain can lead to a more specialized lifestyle in bacterial and eukaryotic lineages (Ochman and Moran 2001; Lawrence 2005). Another example is the obligate ectomycorrhizal symbiont *Amanita bisporigera*, which was found to lack many plant cell-wall-degrading enzymes suggesting that these genes may no longer be required for *A. bisporigera’s* specialized lifestyle (Nagendran et al. 2009).

Genome reduction or retention of a smaller genome is an important evolutionary mechanism that propels divergence of species and more often than not enables adaptation to specific environments. Although smaller genomes are more frequent in prokaryotes, it is not uncommon among eukaryotes including fungal species (Ochman and Moran 2001; Nagendran et al. 2009; Spanu et al. 2010).

### Mobile Genetic Elements

Although *G. morbida* has a smaller genome size, the pathogen possesses a slightly higher percentage of repeats than *G. flava* and *G. putterillii* (table 5). The extent to which mobile genetic elements affect genome evolution in *Geosmithia* is unknown, but mobile genetic elements may be influential drivers of adaptive evolution in *G. morbida*. They are known to be responsible for genomic rearrangements and expansion, horizontal gene transfer and generation of new genes (Casacuberta and González 2013; Stukenbrock and Croll 2014). For example, *Fusarium oxysporum* has a genome nearly 60 Mb in length and contains 16.83 Mb of repetitive sequences (Ma et al. 2010). Although *G. morbida* harbors fewer mobile genetic elements than fungal species such as *F. oxysporum*, it is possible that such elements have contributed to the evolution of pathogenicity in *Geosmithia* via horizontal gene transfer and/or changes in gene numbers. Understanding the role of mobile genetic elements within genus *Geosmithia* may be key in discovering the genetic basis behind the evolution of pathogenicity.

### Repeat-Induced Point Mutations

None of the *Geosmithia* species contain AT-rich regions which are an indicator of repeat-induced point mutations. The paucity of substrate (repetitive genetic elements) could explain the lack of such mutations: if the genome contains low repeats, then repeat-induced point mutations will also occur at a smaller rate.

### Species-Specific Genes

We identified three genes unique to *G. morbida* which are all involved in stress responses that can be induced by the host immune system during the infection process. For example, aldehyde dehydrogenases are part of a large protein family that detoxify aldehydes and alcohols in all organisms including fungal species (Asiimwe et al. 2012). Hydantoinase B/oxoprolinase is involved in the synthesis of glutathione, a compound essential for basic cellular functions but also important in cellular defense against oxidative stress (Pócsi et al. 2004). Glutathione has been shown to chelate damaging metal ions by inhibiting their spread in the cell (Pócsi et al. 2004), and to prevent the accumulation of H_2_O_2_ in *Paxillus involutus* (Ott et al. 2002). Finally, ATP-binding cassette (ABC) proteins belong to an especially large family of proteins that regulates transport of substances across the cellular membrane. In pathogenic fungi, they are involved in drug resistance and in the production of defense molecules (Krattinger et al. 2009; Wang et al. 2013; Karlsson et al. 2015).

### Carbohydrate-Active Enzymes and Peptidases

Although one might expect the pathogen *G. morbida* to possess more carbohydrate binding enzymes than its nonpathogenic relatives, our results indicated that all fungi except *F. solani* have a similar overall distribution (fig. 1). *Fusarium solani* contains more CAZymes than any other pathogen or nonpathogen. This *Fusarium* species is a generalist necrotrophic pathogen that is believed to possess more CAZymes than biotrophic and hemibiotrophic fungi. This discrepancy may be due to the fact that necrotrophic pathogens require an extensive toolkit to promote host cell death as quickly as possible; whereas biotrophs need to keep the host alive, and dispensing large number of degradative enzymes can be detrimental to that aim (Zhao et al. 2013).

In addition to CAZymes, we searched for peptidases. *Geosmithia morbida* has the third highest percent of predicted proteases after *C. militaris* (insect pathogen) and *G. clavigera* (fig. 2 and supplementary table S3, Supplementary Material online). Moreover, *G. flava* and *G. putterillii* have the largest percent of peptidases among the nonpathogenic fungi. All three *Geosmithia* species illustrate similar proteolytic profiles and contain no glutamic and mixed peptidases. These results were expected because all three *Geosmithia* species are closely related. Furthermore, given that these species are plant affiliates (except *C. militaris*), the ability to degrade lignin and cellulose is an important life history trait that is conserved throughout fungal pathogens, but perhaps did not give rise to pathogenicity in *G. morbida*.

### Transmembrane Protein and Effector Genes

Transmembrane proteins are important mediators between a host and its pathogens during microbial invasion. Fungal pathogens either penetrate a surface or enter the host through a wound or opening such as stomata in order to gain access to the nutrients in the plant (Chisholm et al. 2006). Once the infiltration process is completed, pathogens are exposed to host plasma membrane receptors that detect pathogen-associated molecular patterns (PAMP) and induce PAMP-triggered immunity (PTI) to prevent further proliferation of the microbe. Transmembrane proteins expressed by a fungal pathogen are crucial during PTI because they are responsible for suppressing PTI directly or by secreting effector molecules, which contain signal peptides necessary for proper targeting and transport (Chisholm et al. 2006; Boller and He 2009). Our analysis of the 38 proteins under positive selection showed that 11 of these possess at least one or more transmembrane domains. Although nearly 30% of the positively selected genes identified were membrane bound, a similar proportion of nonselected genes in *G. morbida* were membrane associated, indicating this result is not strong evidence that interactions with the host surface are drivers of evolution within *G. morbida*. Among proteins under selection, we found no protein that contained a signal peptide, indicating none of these proteins are secretory.

### Secondary Metabolite Biosynthesis Gene Clusters in *Geosmithia* Species

In comparison to the two closely related species, *A. chrysogenum* and *S. grisellum*, the *Geosmithia* species have much smaller repetoires of secondary metabolite biosynthesis gene clusters, with *G. morbida* having even fewer such gene clusters than *G. putterilli* and *G. flava* (supplementary table S5, Supplementary Material online). *Geosmithia morbida* also has a more specialized ecological niche, associating with only one beetle vector (*P. juglandis*) and a narrow plant host range (*Juglans* species), whereas *G. putterilli* and *G. flava* are associated with a variety of tree species and multiple beetle species (Kolařík and Jankowiak 2013). Having a narrow host range and a smaller arsenal of secondary metabolite biosynthesis gene clusters may be advantageous to *G. morbida* so that fewer genes trigger PTI within the plant and its system does not build immunity rapidly.

### Genes under Positive Selection

Our analyses identified several genes under positive selection in *G. morbida* that encode for proteins that have been implicated in pathogenicity in other fungal pathogens (table 6). For instance, a Cullin3-like protein was predicted to be under positive selection. These proteins belong to a group of structurally similar molecules involved in protein degradation, such as the Skp-Cullin-F-box (SCF) ubiquitin ligase complex (Cardozo and Pagano 2004; Pintard et al. 2004). Furthermore, a ubiquitin-conjugating enzyme (E2) that interacts with cullin3 to prepare substrate for degradation, also had a d*N*/d*S* > 1, indicating that both genes are under positive selection within *G. morbida*. Although little is known regarding the precise functional abilities of these complexes, it is possible these proteins are involved in pathogenicity of *G. morbida*. Previous studies have also implicated ubiquitin ligase complexes in infection and disease development (Duyvesteijn et al. 2005; Han et al. 2007).

Our analysis also revealed a regulatory protein homologous to basic leucine zipper (bZIP) transcription factors was under selection. The bZIP proteins are similar to AP-1 transcription factors and monitor several developmental and physiological processes including oxidative stress responses in eukaryotes (Corrêa et al. 2008). Fungal pathogens such as the rice blast fungus *Magnaporthe oryzae* express AP1-like transcription factor called MoAP1 that contains bZIP domain. MoAP1 is highly active during infection and is translocated from the cytoplasm to the nucleus in response to oxidative stress induced by H_2_O_2_ (Guo et al. 2011). MoAP1 regulates enzymes such as laccase and glutamate decarboxylase that are involved in lignin breakdown and metabolism of γ-aminobutyric acid, respectively (Solomon and Oliver 2002; Baldrian 2005; Janusz et al. 2013). Some of the other positively selected genes include ABC transporter, proteases, proteins involved in apoptosis, and proteins related to DNA replication and repair. As previously mentioned, ABC transporters are important mediators that aid in protection against plant defenses as well as natural toxic compounds (Krattinger et al. 2009; Wang et al. 2013; Karlsson et al. 2015; Lo Presti et al. 2015). Apoptosis or programmed cell death helps establish resistance during host–microbe interactions, helps organisms cope with oxidative environments, and may even be essential for infection (Veneault-Fourrey et al. 2006; Kabbage et al. 2013). In fungal species, proteins involved in DNA replication and repair are essential for the formation and penetration of appressorial structures into the host cell (Son et al. 2016). Only five of the 38 genes with evidence of selection encoded proteins with unknown functions. These positively selected genes may also be involved in the evolution and adaptation of *G. morbida*.

A similar analysis comparaing genes under positive selection in *G. clavigera* and *G. morbida* found only two genes to be under positive selection in both organisms. These findings emphasize that evolutionary forces act differently on divergent populations. All fungal pathogens face dissimilar environmental challenges and associate with different hosts both spatially and temporally. Even closely related organisms can be highly distinct molecularly. For instance, the fungi responsible for the Dutch elm disease—*Ophiostoma ulmi* and *Ophiostoma novo-ulmi—*differ in their genetic composition and virulence despite their strong evolutionary relationship (Brasier 2001; Khoshraftar et al. 2013; Comeau et al. 2015).

In conclusion, our study provides insight into the potential mechanisms that may be behind the evolution of pathogenicity within the genus *Geosmithia*. We also identified a small set of genes that may be contributing to pathogenicity of *G. morbida*. Functional experiments and analyses of the expression levels of these genes during infection as compared to gene expression of a nonpathogen would shed light on the mechanisms influencing pathogen evolution.

## Supplementary Material

Supplementary data are available at *Genome Biology and Evolution* online.

## Supplementary Material

Supplementary Tables and Figures
