# Supplementary figures and images for: Comparative Genomics of Pathogenic and Nonpathogenic Beetle-Vectored Fungi in the Genus Geosmithia
Source: Genome Biol Evol. 2017 Nov 23;9(12):3312–27. doi: 10.1093/gbe/evx242 (PMC5737690; doi:10.1093/gbe/evx242)

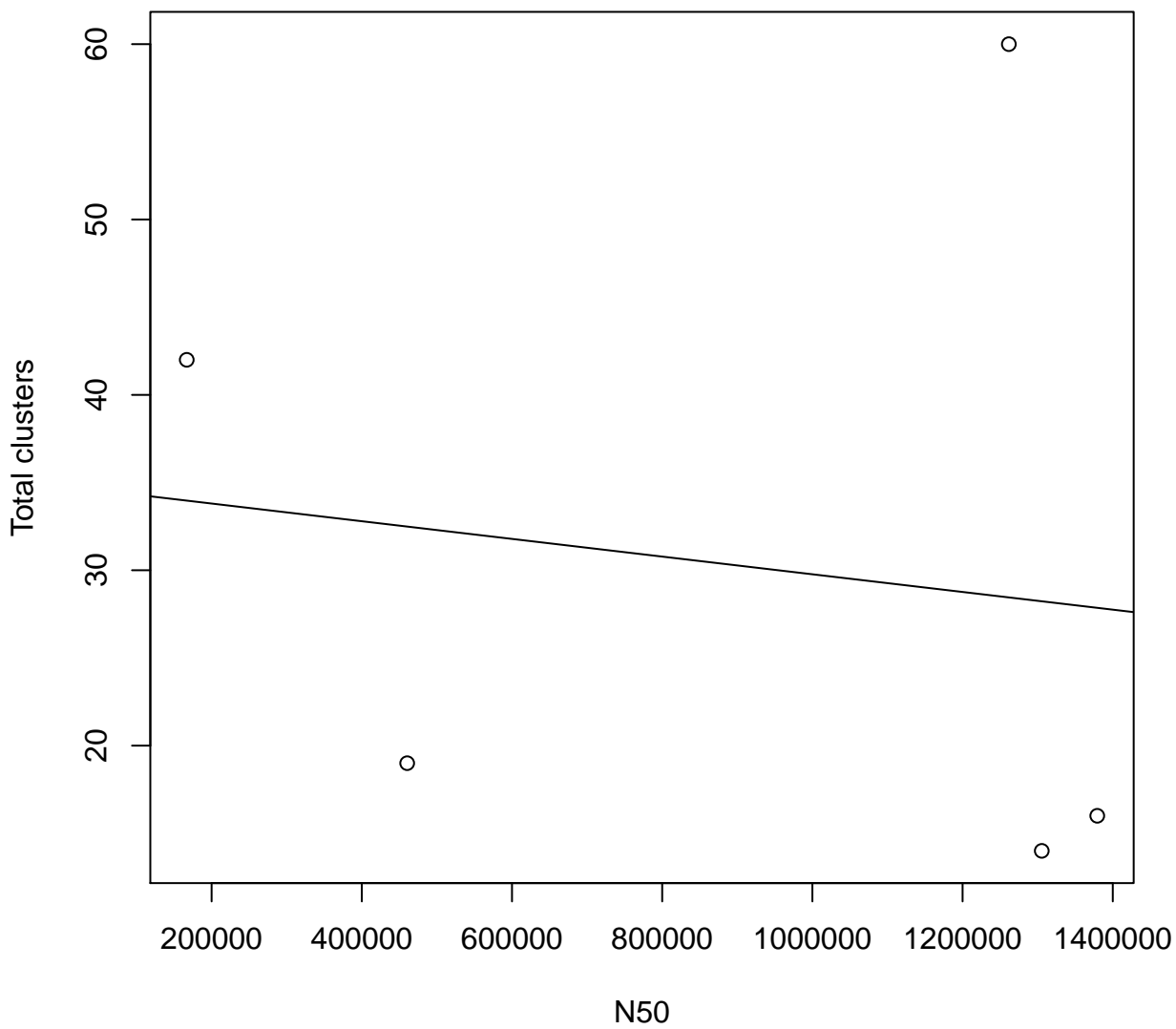

Supplement: Supplementary Tables and Figures [file evx242_supp.zip › Figure_S1_N50_versus_secondaryMetabolites.pdf]

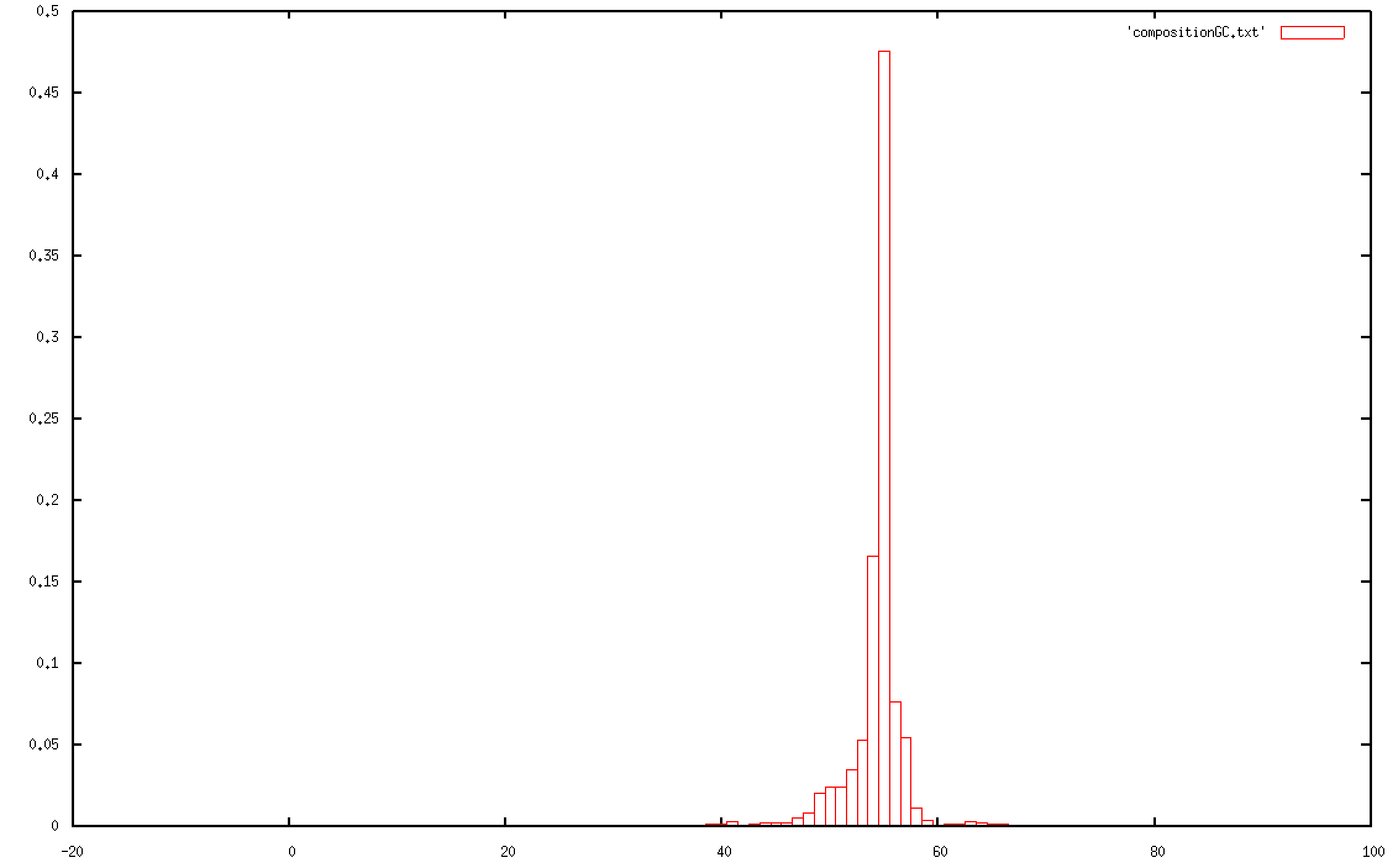

Supplement: Supplementary Tables and Figures [file evx242_supp.zip › Figure_S2_ATcontent_Gmorbida.png]

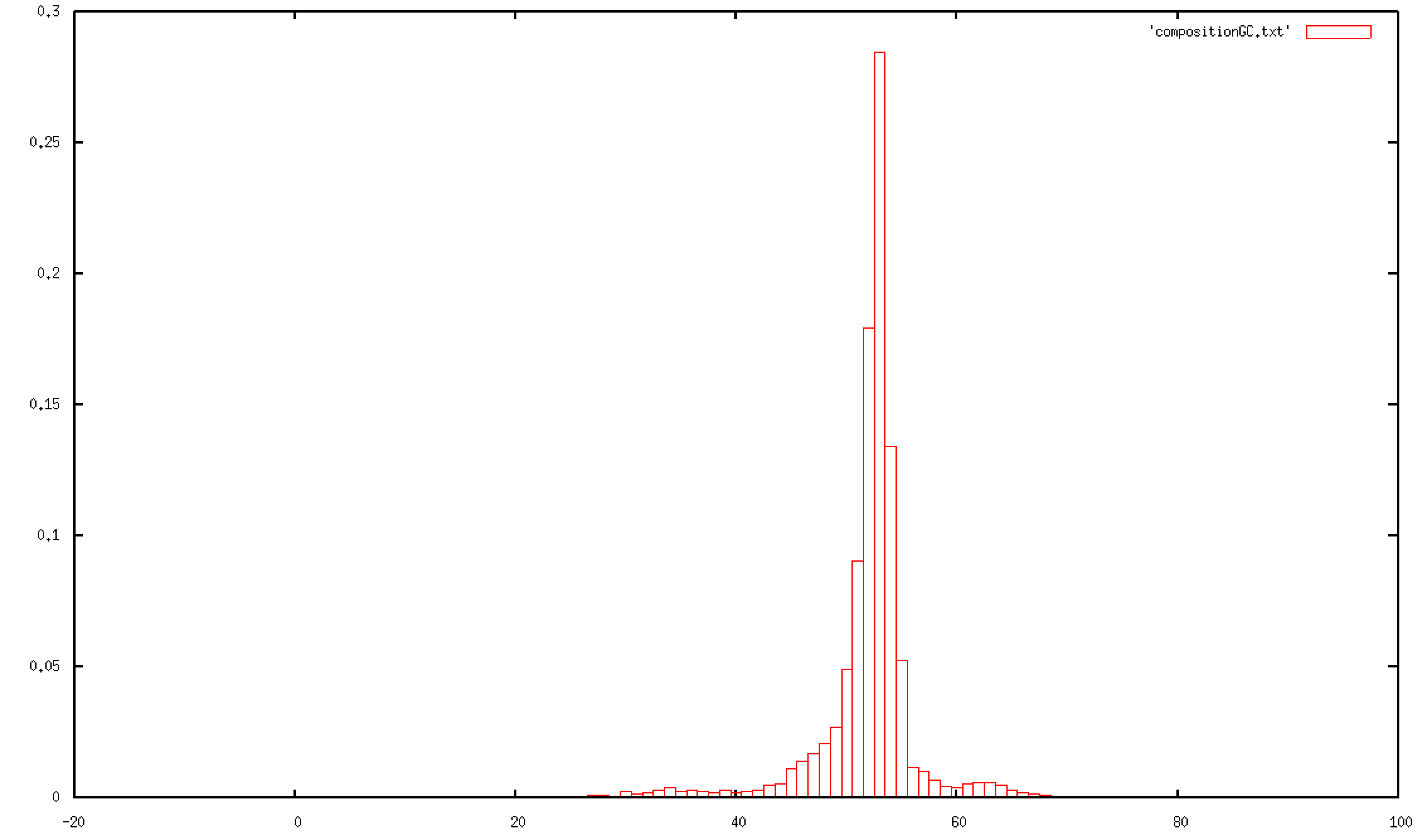

Supplement: Supplementary Tables and Figures [file evx242_supp.zip › Figure_S3_ATcontent_Gflava.png]

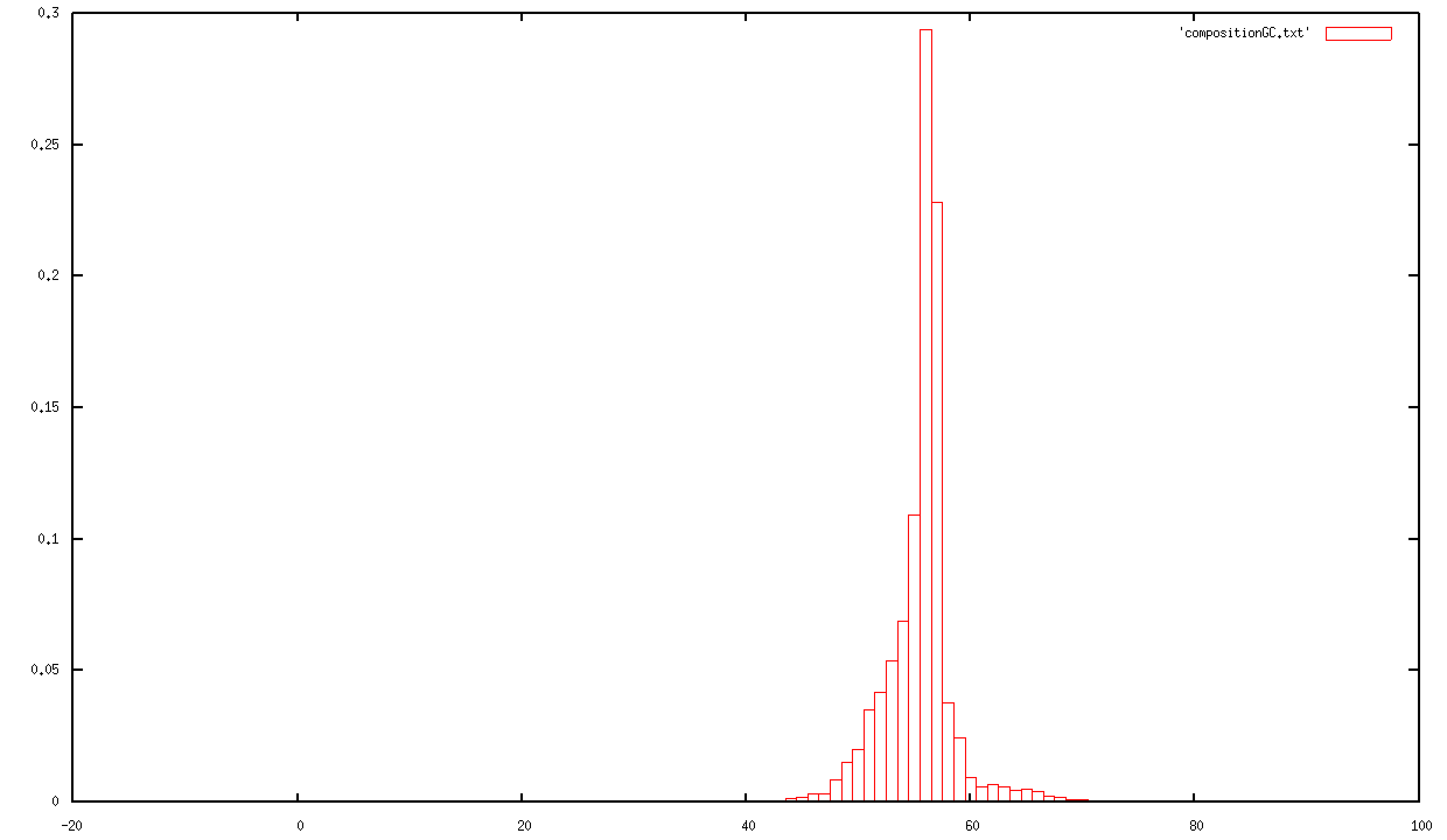

Supplement: Supplementary Tables and Figures [file evx242_supp.zip › Figure_S4_ATcontent_Gputterillii.png]
